# Pristine+™ Everolimus-Eluting Cobalt-Chromium Coronary Stent System: A Prospective, Multicenter Registry

**DOI:** 10.14740/cr2230

**Published:** 2026-07-17

**Authors:** Rishi Sethi, Akshyaya Pradhan, Pravesh Vishwakarma, Prakash Kumar Hazra, Sumanta Chatterjee, Supratip Kundu, Pradip Bhowmik, Parijat Deb Choudhury, Pranav Shamraj, Monika Bhandari, Sridhar Kasturi, Shanivaram Vijay Kumar Reddy, Sheeba George, Yash Paul Sharma, Prashant Kumar Panda, Anil Potdar, Ravi Kumar Patnaik

**Affiliations:** a Department of Cardiology, King George’s Medical University, Lucknow, Uttar Pradesh, India; b Department of Cardiology, Manipal Hospitals - Dhakuria, Kolkata, West Bengal, India; c Department of Cardiology, Manipal Hospitals - Mukundapur, Kolkata, West Bengal, India; d Department of Cardiology, B.K.L. Walawalkar Hospital, Chiplun, Maharashtra, India; e Department of Cardiac Sciences, KIMS Sunshine Hospitals, Hyderabad, Telangana, India; f Department of Cardiology, Jubilee Memorial Hospital, Thiruvananthapuram, Kerala, India; g Department of Cardiology, Postgraduate Institute of Medical Education and Research, Chandigarh, Punjab, India; h Department of Cardiology, One Heart Clinic, Mumbai, Maharashtra, India; i Department of Coronary & Structural Heart Therapies, Relisys Medical Devices Limited, Hyderabad, India

**Keywords:** PCI, Drug-eluting stent, Ultrathin strut, Everolimus, Pristine+™

## Abstract

**Background:**

Recent advances in percutaneous coronary interventions (PCIs) have substantially improved clinical outcomes. Generating real-world evidence on the 1-year outcomes of ultrathin-strut drug-eluting stents (DES) remains important, particularly in healthcare settings where cost and accessibility may influence stent selection.

**Methods:**

A prospective, single-arm, multicenter registry was conducted to evaluate the 1-year safety and efficacy of an indigenous everolimus-eluting cobalt–chromium stent (EES) (Pristine+™ - Make: Relisys Medical Devices Limited, India) featuring an ultrathin strut (60-µm) design. The primary endpoint was the 1-year incidence of major adverse cardiac event (MACE) defined as a composite of cardiac death (CD), myocardial infarction (MI), and stent thrombosis (ST).

**Results:**

The study enrolled 1,088 patients, predominantly male (78.9%). High prevalence of diabetes mellitus (34.7%), hypertension (63.9%), and smoking history (25.7%) was noted. The mean age was 58.6 ± 10.6 years, with 69.9% between 36 and 65 years. A total of 95.9% completed the 1-year follow-up. At 1-year, the Kaplan–Meier incidence of MACE was 2.89% (40 events), comprising 18 CD (1.65%), 13 MI (1.19%), and nine ST (0.83%). All-cause mortality was 2.3% (25 events). One target-vessel myocardial infarction (TV-MI; 0.09%) was reported and no clinically indicated target-lesion revascularization (CI-TLR) events occurred.

**Conclusion:**

This study demonstrated favorable 1-year clinical outcomes with Pristine+™, reflected by low rates of MACE and no CI-TLR during the 1-year follow-up. A total of 1,386 Pristine+ EES were implanted in this study.

## Introduction

Ischemic heart disease (IHD) remains the leading global cause of death, accounting for 9.24 million deaths (108.8 per 100,000) [1]. A recent Bayesian analysis of South Asian Cardiovascular Disease (CVD) reported that this region contributes nearly 60% of the global CVD burden [2]. According to the Global Burden of Disease (GBD) investigators, India’s age-standardized IHD prevalence has been rising by 0.23% per year (95% confidence interval (CI): 0.13–0.32) and is projected to continue increasing through 2040 [2].

With percutaneous coronary interventions (PCIs) now established as a standard treatment for coronary artery disease (CAD), a need to improve accessibility and affordability became evident. Recognizing this challenge, National Pharmaceutical and Pricing Authority (NPPA) of India capped the pricing of drug-eluting stents (DESs) in 2017 to improve affordability and subsequently included them in the national essential medicines list [3]. This intervention represented a significant public-health measure in a country facing a CVD burden, where IHD mortality remains the highest globally [1].

In the current PCI era, physicians and patients can choose from a wide range of indigenous DESs that offer affordable alternatives to United States Food and Drug Administration (US FDA)-approved imported stents. In recent comparisons between these two categories, the indigenous DES demonstrated comparable 1-year rates of death, revascularization and stent thrombosis (ST) [4]. The TALENT study further supported the non-inferiority of an indigenous DES reporting comparable procedural success and clinical outcomes, including low rates of ST among patients with diabetes and multivessel disease [5]. One such indigenous DES is the Pristine+™ everolimus-eluting stent (EES) system, which incorporates an ultrathin strut thickness embedded with a 3-µm coating of biodegradable polymer (BP). It is a new-generation BP-DES that has been in clinical use for over 10 years. A previous study that compared endothelial coverage and vascular recovery across different DESs demonstrated delayed endothelial coverage in sirolimus-eluting stents (SESs), paclitaxel-eluting stents, and zotarolimus-eluting stents compared with EESs [6]. While these mechanistic findings provide useful context, direct assessment of endothelial coverage was not within the scope of the present registry. Further real-world evidence on contemporary EES designs—particularly those used widely in India—is needed to guide DES selection for India’s growing CAD population. Therefore, this prospective, multicenter, post-marketing registry was undertaken to evaluate the 1-year clinical efficacy and safety of Pristine+™ EES in an Indian cohort.

## Materials and Methods

### Study device

**Figure 1:**
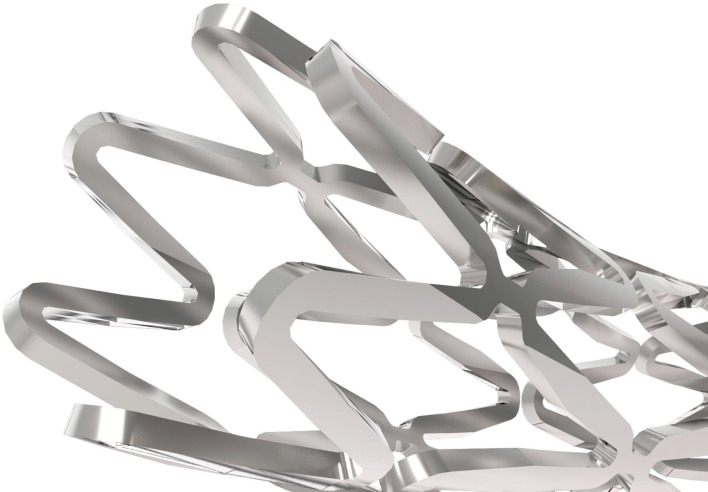
Device design of Pristine+™ everolimus-eluting coronary stent system (Relisys Medical Devices Limited).

Pristine+™ EES is a Drug Controller General of India (DCGI)-approved (2015), ultrathin-strut (60-µm) cobalt–chromium (Co-Cr) coronary stent system based on the L605 Co-Cr platform and consisting of an open and closed-cell architecture (Fig. 1). The design is engineered for improved deliverability and trackability through tortuous or narrowed coronary vessels, with reduced acute recoil (< 4%) and minimal foreshortening (< 2%) after balloon expansion [7]. Drug release is facilitated by a 3-µm polylactic-co-glycolic acid (PLGA) BP coating that enables uniform everolimus distribution over a controlled elution period of approximately 90–120 days [7]. The stent delivery catheter includes radiopaque markers and a hydrophilic coating to aid navigation and accurate DES placement. Pristine+™ is available in lengths of 8–48 mm and diameters of 2.25–5.0 mm.

### Ethical statements

The screening and enrollment procedures were initiated after obtaining approval from respective Institutional Ethics Committees. The study was registered with Clinical Trials Registry – India (CTRI), under Registration Number CTRI/2021/01/030701. All study procedures adhered to the Declaration of Helsinki, Good Clinical Practice (GCP) guidelines, and applicable regulatory requirements.

### Study design

A prospective, multicenter, post-marketing registry of Pristine+™ EES was initiated to evaluate 1-year safety and efficacy outcomes in adults with CAD including those presenting with acute coronary syndromes (ACSs). Data were collected using standardized case report forms completed by trained study personnel at each participating site. Patients were enrolled between January 2021 and December 2023. During the study period, 1,200 patients with CAD were screened for eligibility.

### Inclusion criteria

Patients aged 18–75 years with life expectancy > 1 year including male and non-pregnant, non-lactating female patients, were eligible for enrollment. Eligible patients with *de novo* coronary lesions or prior PCI in non-target vessels were permitted; however, target-lesion in-stent restenosis (ISR) was excluded as described under exclusion criteria.

Baseline evaluation included clinical assessment of angina or ischemia, medical history, and routine laboratory investigations (complete blood count (CBC)), electrocardiography (ECG), serum creatinine, along with 2D echocardiography when indicated. Screening diagnostic tests such as stress testing were performed at the investigator’s discretion.

Lesions with reference vessel diameter of 2.5–4.0 mm were eligible for treatment. Patients were required to comply with scheduled 6- and 12-month follow-up visits.

### Exclusion criteria

Patients with bifurcation lesions involving a side branch > 2 mm or requiring overlapping stents > 60 mm were excluded. Additional exclusions included allergy to clopidogrel, cobalt–chromium, everolimus or contrast media; vessel diameter < 2.5 mm; chronic total occlusions (CTOs), heart failure, prior cerebrovascular events, ISR, advanced chronic kidney disease requiring dialysis, chronic organ failure requiring ongoing specialist management, major systemic disease, heart transplantation, or a major bleeding episode within 6 months.

### Study procedures and complication management

Anticoagulant therapy was administered as per standard periprocedural and antiplatelet guidelines, using either low-molecular-weight or unfractionated heparin to maintain recommended activated clotting time (ACT) ranges.

Balloon pre-dilatation, post-dilatation, and DES sizing followed routine PCI standards, with the target of achieving thrombolysis in myocardial infarction (TIMI) grade 3 flow. Bailout measures—including additional stenting–were performed for major dissections, abrupt vessel closure, or ischemic deterioration.

Aspirin and dual antiplatelet therapy (DAPT) with clopidogrel, prasugrel, or ticagrelor were provided according to contemporary guidelines [8], with transition to single purinergic receptor (P2Y12) inhibitor therapy after the standard DAPT period in appropriate patients.

Lesions were classified as per American College of Cardiology/American Heart Association (ACC/AHA) criteria [9]. Peri-procedural events were managed by the heart team with continuous monitoring, and post-procedural care ensured early detection and management of bleeding or ischemic complications prior to catheterization-lab discharge.

### Endpoints

The primary endpoint was the 12-month MACE rate, defined as the composite of cardiac death (CD), myocardial infarction (MI), and ST. Secondary endpoints included target-lesion failure (TLF), defined as the composite of CD, target-vessel MI (TV-MI), and clinically indicated target-lesion revascularization (CI-TLR), and procedural and device success.

### Definitions of clinical endpoints

Clinical endpoints were defined according to the Academic Research Consortium (ARC-2) criteria [10]: CI-TLR: repeat revascularization of the index lesion performed in the presence of ≥ 50% stenosis with ischemic symptoms or objective evidence of ischemia attributable to the target-lesion. MI: defined as per ARC-2 criteria, including biomarker elevation with ischemic symptoms or ECG/imaging changes. TLF: a composite of CD, TV-MI, and CI-TLR. ST was classified as acute (≤ 24 h), subacute (> 24 h to 30 days), late (> 30 days to 1 year), or very late (> 1 year). Clinical endpoints were assessed at 30 days, 6 months, and 12 months through clinical or telephonic follow-up. Procedural success was defined as achieving < 30% residual in-stent stenosis (visual estimate), and no in-hospital MACE during the 3–7 days index hospitalization. In-hospital deaths were counted as failures under procedural success. Device success was defined as successful delivery and deployment of the assigned stent with < 30% final residual stenosis (visual estimate). Although device success forms part of procedural success, they are reported separately to distinguish device-related technical performance from overall procedural outcomes. Procedural and device success were analyzed at lesion level, whereas clinical outcomes were analyzed at patient level. Bleeding events were recorded as part of routine adverse event monitoring during follow-up; however, no standardized bleeding classifications were prespecified, thereby limiting the interpretability of bleeding-related findings.

### Statistical analysis

All analyses were performed on Microsoft Excel (Microsoft Corporation, USA) and R software (R Foundation for Statistical Computing, Vienna, Austria). Baseline characteristics were summarized as frequencies and percentages for categorical variables and mean ± standard deviation (SD) for continuous variables. Kaplan–Meier estimates were used to construct cumulative event curves for MACE and TLF. The sample size was not pre-determined as this post-marketing registry enrolled all consecutive eligible patients during the predefined study period. Analyses were conducted using an intention-to-treat (ITT) approach.

## Results

**Figure 2:**
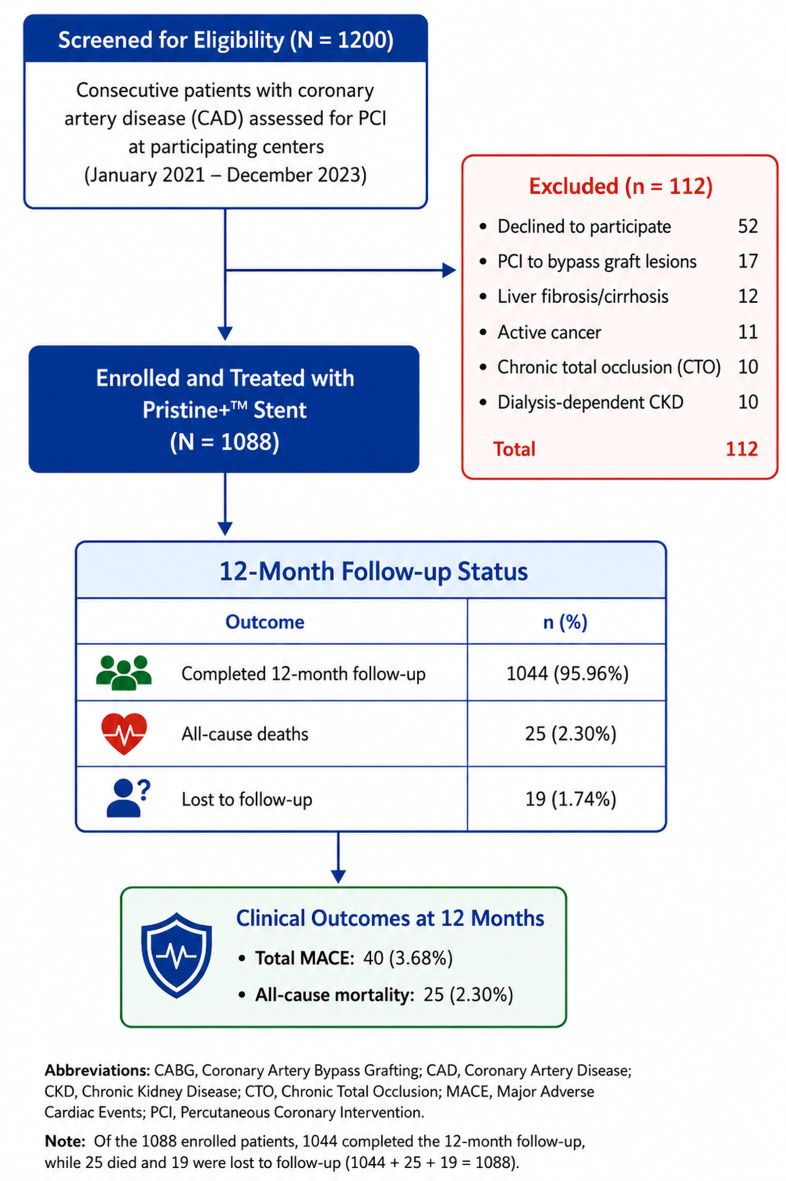
Patient flow of the Pristine+™ Registry according to STROBE guidelines.

A total of 1,200 consecutive patients with CAD who were considered for treatment with the Pristine+™ EES at participating centers during the study period were screened for eligibility, of whom 1,088 were enrolled in the registry. These patients do not represent all PCI procedures performed at the participating institutions. Overall follow-up completion was high, with 1,044 patients (95.96%) completing the 12-month visit (Fig. 2). Nineteen (1.74%) patients were lost to follow-up while 112 patients (9.33%) were excluded for not meeting eligibility criteria. Figure 2 provides the number of patients excluded for each eligibility criterion.

### Baseline characteristics

The overall study population had a mean age of 58.6 ± 10.6 years with a predominance of male patients (n = 859, 78.9%). Comorbidities included diabetes (n = 378, 34.7%), hypertension (n = 695, 63.9%), and smoking history (n = 280, 25.7%). Mean body mass index (BMI) was 24.1 kg/m^2^. Long standing diabetes (> 10 years) was present in 124 patients (11.4%). Prior revascularizations included coronary artery bypass surgery (CABG) in 21 patients (1.93%) and PCI in 55 patients (5.05%).

The majority of patients (95.3%) presented with ACS (n = 1,037; Table 1), while patients with chronic coronary syndrome (CCS) comprised 4.7% (n = 51). Although CCS patients did not have CTOs, only clinically significant lesions deemed suitable for PCI were included in the registry. Consequently, the small number of CCS patients represented a selected population with angiographically significant coronary stenoses, which may account for the observed absence of TIMI grade 3 flow at presentation.

**Table 1:** Baseline and at Discharge Characteristics (N = 1,088 Patients)

Category	Details
Demographics	
Male	859 (78.9%)
Female	229 (21.1%)
Age	58.6 ± 10.6 years
BMI	24.1 ± 3.5 kg/m^2^
Comorbidities	
Diabetes	378 (34.7%)
> 10 years	124 (11.4%)
Hypertension	695 (63.9%)
CKD	143 (13.4%)
Smokers	280 (25.7%)
Cardiac function	
LVEF overall	49.1 ± 10.0
< 40	149 (13.7%)
40–50	542 (49.8%)
> 50	397 (36.5%)
Vital signs	
Systolic BP	130.1 ± 10.4 mm Hg
Diastolic BP	83.4 ± 8.7 mm Hg
Heart rate	80.0 ± 14.2 bpm
Prior cardiac history	
PTCA	55 (5.1%)
MI	81 (7.4%)
CABG	21 (1.9%)
Clinical presentation	
ACS	1,037 (95.3%)
STEMI	617 (56.7%)
NSTEMI	117 (10.8%)
Unstable angina	303 (27.9%)
CCS	51 (4.7%)
Pharmacotherapy at discharge	
Antiplatelets: aspirin	1,078 (99.1%)
Ticagrelor	970 (89.2%)
Clopidogrel	80 (7.4%)
Prasugrel	22 (2.0%)
Statins	1,068 (98.2%)
Beta-blockers	628 (57.7%)
Calcium channel blockers (CCBs)	125 (11.5%)

ACS: acute coronary syndrome; BMI: body mass index; BP: blood pressure; CABG: coronary artery bypass grafting; CCBs: calcium channel blockers; CCS: chronic coronary syndrome; CKD: chronic kidney disease; LVEF: left ventricular ejection fraction; MI: myocardial infarction; NSTEMI: non-ST-segment elevation myocardial infarction; N: number of patients; PTCA: percutaneous transluminal coronary angioplasty; P2Y12: purinergic receptor P2Y12; SD: standard deviation; STEMI: ST-segment elevation myocardial infarction.

The study population had a mean left ventricular ejection fraction (LVEF) of 49.1±10.0%. Nearly half of the patients (49.8%) had mildly reduced LVEF (40–50%), while 13.7% had severely impaired ventricular function (LVEF < 40%) and 36.5% had preserved LVEF (> 50%) (Table 1).

Across 1,088 patients, a total of 1,386 target-lesions were treated during the index PCI procedure with the Pristine+™ stent system. Lesion distribution was left anterior descending (LAD) artery (47.6%, n = 660), right coronary artery (RCA) (32.1%, n = 445), and left circumflex coronary artery (LCx) (12.8%, n = 178) (Table 2).

**Table 2:** Procedural Characteristics (N = 1,088 Patients; 1,386 Lesions)

Category	Details
Lesion classification (ACC/AHA)	
Type A	239 (17.2%)
Type B1	446 (32.2%)
Type B2	229 (16.5%)
Type C	174 (12.6%)
Lesion locations (1,386 lesions)	
LAD	660 (47.6%)
RCA	445 (32.1%)
LCx	178 (12.8%)
LMCA	14 (1.0%)
Distal branches	64 (4.6%)
Other	25 (1.8%)
Stent lengths (mm)	
15	26 (1.9%)
18	182 (13.1%)
20	217 (15.7%)
24	255 (18.4%)
28	316 (22.8%)
32	390 (28.1%)
Stent diameters (mm)	
2.5	76 (5.5%)
2.75	267 (19.3%)
3.0	649 (46.8%)
3.5	380 (27.4%)
4.0	14 (1.0%)
TIMI flow pre-PCI	
TIMI 0	108 (9.9%)
TIMI 1	234 (21.5%)
TIMI 2	746 (68.6%)
TIMI flow post-PCI	
TIMI 2	27 (2.5%)
TIMI 3	1,061 (97.5%)
Procedural success	1,083 (99.5%)
Device success	1,074 (98.7%)
Complications	
Severe dissection	2
Abrupt closure	1
In-hospital deaths	2

Lesion location, stent length, and stent diameter are reported at the lesion level (1,386 lesions treated). Lesion classification, TIMI flow, procedural success, and device success are reported at the patient level based on the index target lesion (N = 1,088). ACC/AHA: American College of Cardiology/American Heart Association; LAD: left anterior descending artery; LMCA: left main coronary artery; LCx: left circumflex artery; mm: millimeter; PCI: percutaneous coronary intervention; RCA: right coronary artery; TIMI: thrombolysis in myocardial infarction.

### Procedure related

As seen in Table 2, most procedures were performed via radial approach (n = 934, 85.8%) with 14.2% (n = 154) performed femorally. At presentation, the myocardial flow was completely occluded in 9.93% (n = 108) patients (TIMI 0 flow) and 746 (68.57%) patients presented with TIMI 2 (Table 3).

**Table 3:** Cumulative 12-Month Outcomes

Category	Details
Mortality	
Cardiac deaths	18 (1.65%)
All-cause deaths	25 (2.3%)
Male deaths	16 (64%)
Female deaths	9 (36%)
Ischemic events	
MI	13 (1.19%)
TV-MI	1 (0.09%)
CI-TLR	0
Stent thrombosis	
Total	9 (0.83%)
Probable	6 (0.55%)
Definite	3 (0.28%)
Late	0
Composite endpoints	
MACE	40 (2.89%)
TLF	19 (1.77%)
Safety	
Major bleeding	1 (0.09%)
Pharmacotherapy at 12 months	
Aspirin	1,023 (94.0%)
Ticagrelor	726 (66.7%)
Clopidogrel	141 (13.0%)
Prasugrel	19 (1.7%)
Statins	1,027 (94.4%)
Beta-blockers	704 (64.7%)
CCBs	122 (11.2%)

*Post hoc sex-stratified all-cause mortality. CCBs: calcium channel blockers; CI-TLR: clinically indicated target-lesion revascularization; MACE: major adverse cardiac event; MI: myocardial infarction; N: number of patients; ST: stent thrombosis; TLF: target-lesion failure; TV-MI: target vessel-attributed myocardial infarction.

**Figure 3:**
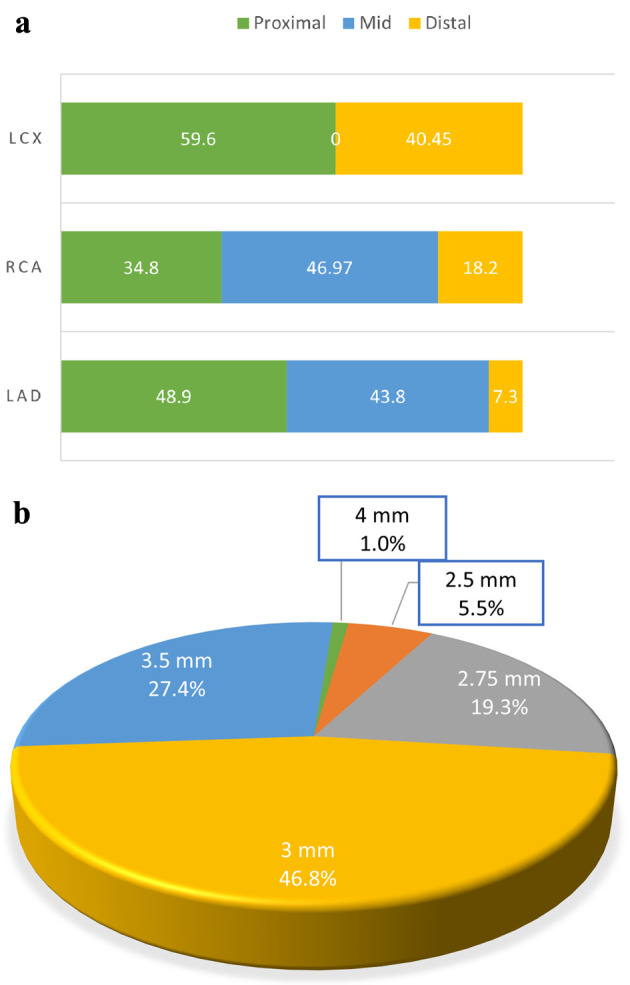
(a) Distribution of lesions in the main coronaries. (b) Pristine+™ EES stent diameters used. EES: everolimus-eluting stent.

Details of the stent diameter and stent length are presented in Table 2 and Figure 3.

### Clinical outcomes

Post-procedure, TIMI grade 3 flow was achieved in 1,061 patients (97.4%) (Table 2). No patient required bail-out CABG. Procedural success was 99.5% (n = 1,083) with five patients (0.46%) not meeting this endpoint due to severe dissection (n = 2), abrupt vessel closure (n = 1), and in-hospital deaths (n = 2). Device success was 98.7% (n = 1,074) with 14 patients (1.9%) not meeting this endpoint due to residual stenosis > 30% despite optimal stent deployment (Table 2). Importantly, in-hospital deaths were counted as failures under procedural success but not under device success, which was defined strictly by angiographic criteria.

Pharmacotherapy at discharge and at 12 months (antiplatelet agents and other cardiac medications) are shown in Tables 1 and 3.

At 12 months, the Kaplan–Meier cumulative incidence of MACE was 2.89% (n = 40), comprising CD (1.65%, n = 18) (definite: 5; possible: 13), MI (1.19%, n = 13), and ST (0.83%, n = 9) (definite: 0.28%, n = 3; probable: 0.55%, n = 6).

**Figure 4:**
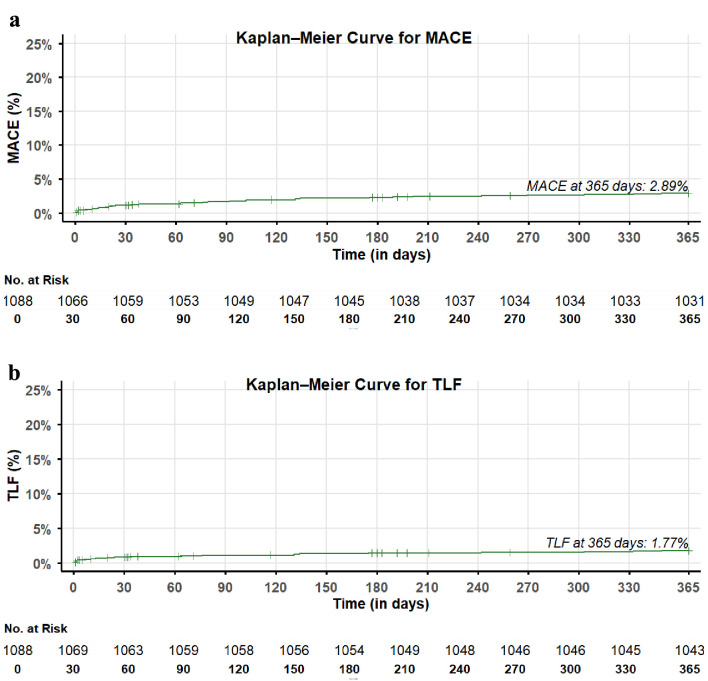
The 12-month Kaplan–Meier cumulative incidence curve for (a) MACE and (b) TLF. MACE: major adverse cardiac event; TLF: target lesion failure.

TLF occurred in 1.77% (n = 19) and included one event (0.09%) TV-MI and 0 event CI-TLR. *Post hoc* sex-stratified mortality showed female of 36% (n = 9) and male of 64% (n = 16). The Kaplan–Meier analysis showed cumulative incidence of MACE of 2.89% and cumulative incidence of TLF of 1.77%. There were no late ST events. MACE occurrence declined substantially after the first 3 months post-PCI (Fig. 4).

Major bleeding events were rare, occurring in 0.09% (n = 1) of patients. No life-threatening bleeding was observed during the 12-month follow-up.

### Clinical outcomes (subgroup analysis)

Subgroup analyses demonstrated generally low 12-month rates of CD, MI, ST, and TLF across major clinical groups. As expected, patients with CKD exhibited the highest event rates among the evaluated subgroups (CD: 4.2%, MI: 2.1%, ST: 1.4%, and MACE: 6.3%), reflecting the well-established higher cardiovascular risk associated with renal dysfunction (Table 4).

**Table 4:** Clinical Outcome Comparison (CKD, Diabetes, Hypertension, Smokers Subsets)

Outcome	CKD (N = 143)	Diabetes (N = 378)	Hypertension (N = 695)	Smokers (N = 280)
Cardiac death	6 (4.2%)	9 (2.4%)	12 (1.7%)	5 (1.8%)
MI	3 (2.1%)	4 (1.1%)	13 (1.9%)	2 (0.7%)
Stent thrombosis	2 (1.4%)	7 (1.9%)	6 (0.9%)	2 (0.7%)
MACE	9 (6.36%)	13 (3.48%)	25 (3.67%)	7 (2.5%)
CI-TLR	0	0	0	0
TV-MI	0	0	1 (0.1%)	0
TLF	6 (4.24%)	9 (2.4%)	13 (1.91%)	5 (1.8%)

CI-TLR: clinically indicated target-lesion revascularization; CKD: chronic kidney disease; MI: myocardial infarction; MACE: major adverse cardiac events; N: number of patients; ST: stent thrombosis; TLF: target-lesion failure; TV-MI: target vessel myocardial infarction.

**Figure 5:**
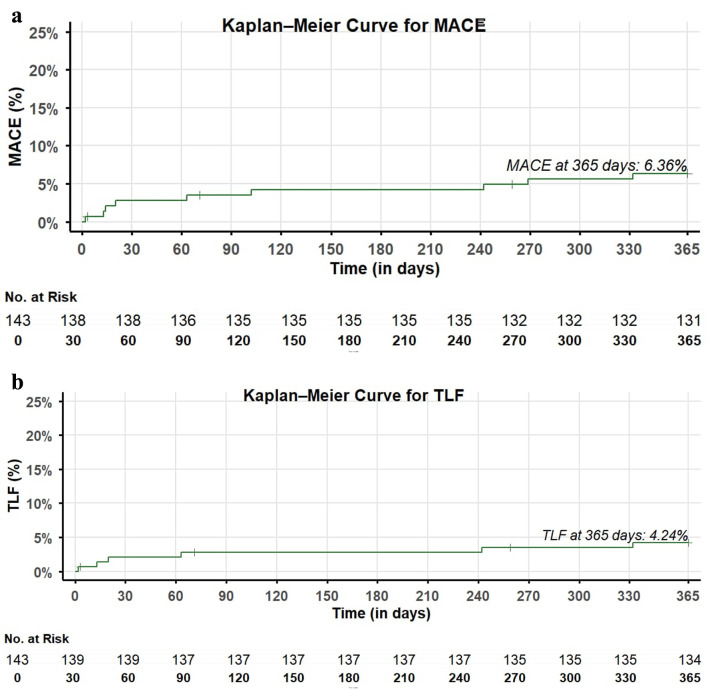
CKD: present Kaplan–Meier analyses of subgroup cumulative incidence for (a) MACE and (b) TLF. CKD: chronic kidney disease; MACE: major adverse cardiac event; TLF: target lesion failure.

**Figure 6:**
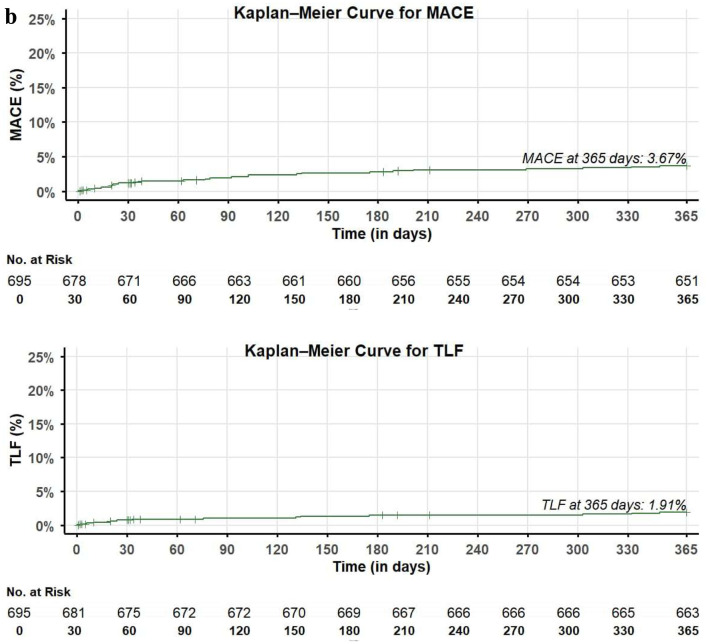
Hypertension: present Kaplan–Meier analyses of subgroup cumulative incidence for (a) MACE and (b) TLF. MACE: major adverse cardiac event; TLF: target lesion failure.

**Figure 7:**
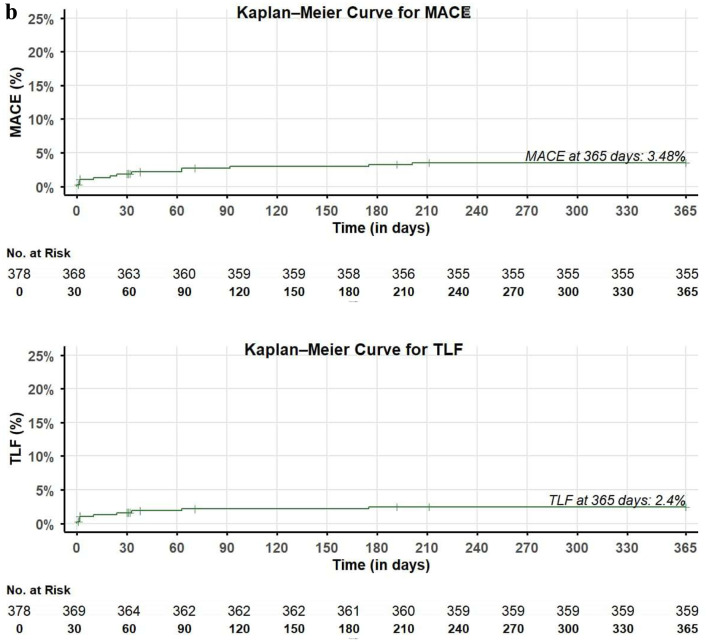
Diabetes: present Kaplan–Meier analyses of subgroup cumulative incidence for (a) MACE and (b) TLF. MACE: major adverse cardiac event; TLF: target lesion failure.

Figures 5–7 show the Kaplan–Meier analyses of subgroup cumulative incidence of MACE and TLF.

## Discussion

Herein, we report the clinical outcomes from a 1-year safety and performance evaluation of indigenous Pristine+™ EES in a nationwide cohort comprising predominantly of ACS patients. This registry achieved a 12-month follow-up completion rate of 95.7%. Procedural outcomes were favorable, with high procedural and device success. At 12 months, the cumulative incidence of MACE was 2.89%. Despite comorbidities, major bleeding events were rare. ACS represents one of the most clinically challenging patient populations, characterized by an overall elevated risk of long-term adverse cardiac events. Despite the inclusion of 1,037 ACS patients, the outcomes with Pristine+ remained favorable, demonstrating low rates of MACE at 12 months. However, as a single-arm study, these findings should be interpreted as descriptive rather than comparative.

Evidence from prior studies of EESs has shown favorable vascular healing characteristics compared with earlier-generation DES [11]. The present registry contributes real-world data from an Indian CAD population. Although comparisons with published trials (e.g., Xience, Orsiro, Firehawk, Synergy) provide contextual reference, differences in study design, patient selection, and follow-up duration preclude any direct inference of relative performance [12–15] (Table 5). The observed cardiac mortality rate of 1.65% was numerically higher than that reported in several contemporary DES registries; however, the present study enrolled a substantially higher-risk population, with 95.3% ACS and 56.7% STEMI patients. In contrast, several comparator studies included predominantly stable CAD patients with lower baseline cardiovascular risk. Therefore, differences in mortality are more likely attributable to variations in patient risk profiles rather than stent-related performance, and cross-trial comparisons should be interpreted with caution.

**Table 5:** Comparison of Outcomes From Contemporary Sirolimus-Eluting and Everolimus-Eluting Stents

Trial	Device	Population	ACS status	Cardiac death (%)	MI/TV-MI (%)	TLF (%)	TLR (%)	ST (%)
meriT-V (Abizaid, 2023) [15]	Xience (EES)	86 *de novo* CAD	Stable CAD	0	6	NR	2.4	1.2
BIOSTEMI (Pilgrim, 2021) [12]	Orsiro (BP-SES)	649 STEMI	ACS (100% STEMI)	2.9	1.5 (TV-MI)	5.1	2.5	1.4
BIOSTEMI (Pilgrim, 2021) [12]	Xience (EES)	651 STEMI	ACS (100% STEMI)	3.2	2.0 (TV-MI)	8.1	5.1	1.8
TARGET All-Comers (Xu, 2019) [13]	Firehawk (BP-SES)	823 CAD (ACS + ISR)	Mixed	1.7	5.7 (TV-MI)	8.7	2.6	1.7

Cross-trial comparisons should be interpreted cautiously because of differences in study design, patient characteristics, ACS prevalence, endpoint definitions, and follow-up methodology. ACS: acute coronary syndrome; CAD: coronary artery disease; EES: everolimus-eluting stent; STEMI: ST-segment elevation myocardial infarction; BP-SES: biodegradable polymer sirolimus-eluting stent; TVF: target vessel failure; TLF: target-lesion failure; TLR: target lesion revascularization; MI: myocardial infarction; ISR: in-stent restenosis.

Notably, no CI-TLR was observed within 12 months, and no late ST occurred during the same period.

The ultrathin-strut cobalt-chromium platform with BP reflects current-generation EES design [8, 11], though mechanistic effects cannot be determined from this registry. Nonetheless, the low rate of ST within 12 months is reassuring.

Among the nine ST events, six were classified as probable and three as definite. The probable cases presented as CD or hemodynamic compromise without angiographic confirmation, and therefore no repeat revascularization was performed. In the definite cases, patients either did not undergo target-lesion revascularization or were managed medically based on clinician’s assessment. Consequently, no CI-TLR events were recorded. All ST events–whether probable or definite–were included in the cumulative endpoint analyses.

Subgroup analyses including patients with diabetes, CKD, hypertension, and smoking history demonstrated numerically low event rates, suggesting that baseline comorbidity did not adversely influence short-term safety outcomes. Antiplatelet management followed guidelines, with patients maintained on DAPT for 3–6 months before transition to single P2Y12 inhibitor therapy.

Pharmacotherapy trends including the use of statins, calcium-channel blockers, and beta-blockers (BBs) showed sustained optimization through 12-month follow-up, reflecting adherence to guideline-recommended secondary prevention. The rising cardiovascular risk burden among Indian CAD patients underscores the importance of real-world evidence from diverse clinical settings [16].

The high prevalence of cardiovascular risk factors observed in this registry is consistent with previous reports demonstrating that South Asians experience CAD and MI at a younger age and carry a substantial burden of diabetes, smoking, obesity, and other cardiometabolic risk factors [17, 18].

Stent choice in CCS typically allows for shared decision making, whereas in ACS the decision is time-critical. As our registry predominantly included ACS patients, the findings reflect this urgent clinical context.

Overall, the outcomes observed in this registry likely reflect multiple factors, including appropriate patient selection, adherence to periprocedural pharmacotherapy standards, and consistent follow-up practices across participating centers. While these findings provide useful insights for clinical practice, confirmation through randomized controlled trials with longer follow-up is needed to assess comparative performance and long-term safety.

### Limitations

As this was a single-arm study with broad eligibility criteria, the potential for selection bias cannot be fully excluded. The absence of a comparator arm also limits the objectivity of the study outcomes, and the results should therefore be interpreted as observational rather than comparative. This registry was designed to evaluate the performance of the study stent in managing both ACS and CCS across a wide clinical spectrum, including patients with prior revascularization; however, the lack of protocol-mandated angiographic follow-up restricts the ability to draw conclusions regarding mechanistic endpoints such as restenosis or healing patterns. Future studies incorporating angiographic or intravascular imaging endpoint analysis would help substantiate these findings and offer stronger support for real-time clinical decision making.

Although the registry provides valuable real-world evidence on PCI outcomes using this DES, the generalizability of the findings may be limited because patients with significant comorbidities were excluded as part of the study protocol. Additionally, procedural data such as intravascular imaging and debulking device utilization were not uniformly collected across sites, which may influence interpretation of lesion-level outcomes.

Nevertheless, this study offers meaningful insights into contemporary PCI practice patterns and clinical outcomes with this DES in an Indian population. Clinicians may also consider conducting meta-analyses of contemporary DES systems to contextualize these results within the broader EES class and to strengthen comparative insights across multiple devices.

### Conclusions

The Pristine+™ EES demonstrated favorable 12-month clinical outcomes, with low rates of TLF, MACE, and no late ST in this real-world registry. A total of 1,386 Pristine+ EES were implanted in this study.

## Data Availability

The source data used for reporting the findings of this study can be availed from the corresponding author upon reasonable requests.

## Abbreviations

ACC: American College of Cardiology
ACS: acute coronary syndrome
ACT: activated clotting time
AHA: American Heart Association
ARC: Academic Research Consortium
BBs: beta-blockers
BMI: body mass index
CCS: chronic coronary syndrome
CABG: coronary artery bypass graft
CAD: coronary artery disease
CBC: complete blood count
CCBs: calcium channel blockers
CD: cardiac death
CKD: chronic kidney disease
CI: confidence interval
CTO: chronic total occlusion
CTRI: Clinical Trials Registry – India
CVD: cardiovascular disease
GBD: Global Burden of Disease
PMS: post-marketing surveillance
DAPT: dual antiplatelet therapy
DCGI: Drugs Controller General of India
DES: drug-eluting stent
EES: everolimus-eluting stent
ECG: electrocardiography
ISR: in-stent restenosis
IHD: ischemic heart disease
ITT: intention-to-treat
LAD: left anterior descending artery
LAR: legally accepted representative
LFU: lost to follow-up
LMCA: left main coronary artery
LCx: left circumflex artery
LVEF: left ventricular ejection fraction
MACE: major adverse cardiac event
MI: myocardial infarction
NSTEMI: non-ST-segment elevation myocardial infarction
N: number of patients
mm: millimeter
OCT: optical coherence tomography
PCI: percutaneous coronary intervention
PLGA: polylactic-co-glycolic acid
PTCA: percutaneous transluminal coronary angioplasty
P2Y12: purinergic receptor P2Y12
RCA: right coronary artery
RV: right ventricle
SAE: serious adverse event
SES: sirolimus-eluting stent
SoC: standard of care
ST: stent thrombosis
STEMI: ST elevation myocardial infarction
TIMI: thrombolysis in myocardial infarction
TVF: target vessel failure
TVR: target vessel revascularization
TLF: target-lesion failure
TLR: target-lesion revascularization
UA: unstable angina
